# Supplementary material for: The Microbiome of an Outpatient Sports Medicine Clinic During a Global Pandemic: Effects of Implementation of a Microbiome-Specific Cleaning Program
Source: Microorganisms. 2025 Mar 25;13(4):737. doi: 10.3390/microorganisms13040737 (PMC12029496; doi:10.3390/microorganisms13040737)
Supplement: Supplementary file 1 [file microorganisms-13-00737-s001.zip › microorganisms-3501210-supplementary.pdf]

Data Supplement, Figure S1: Clinic Floor Plan and Examples of Probe on Surfaces

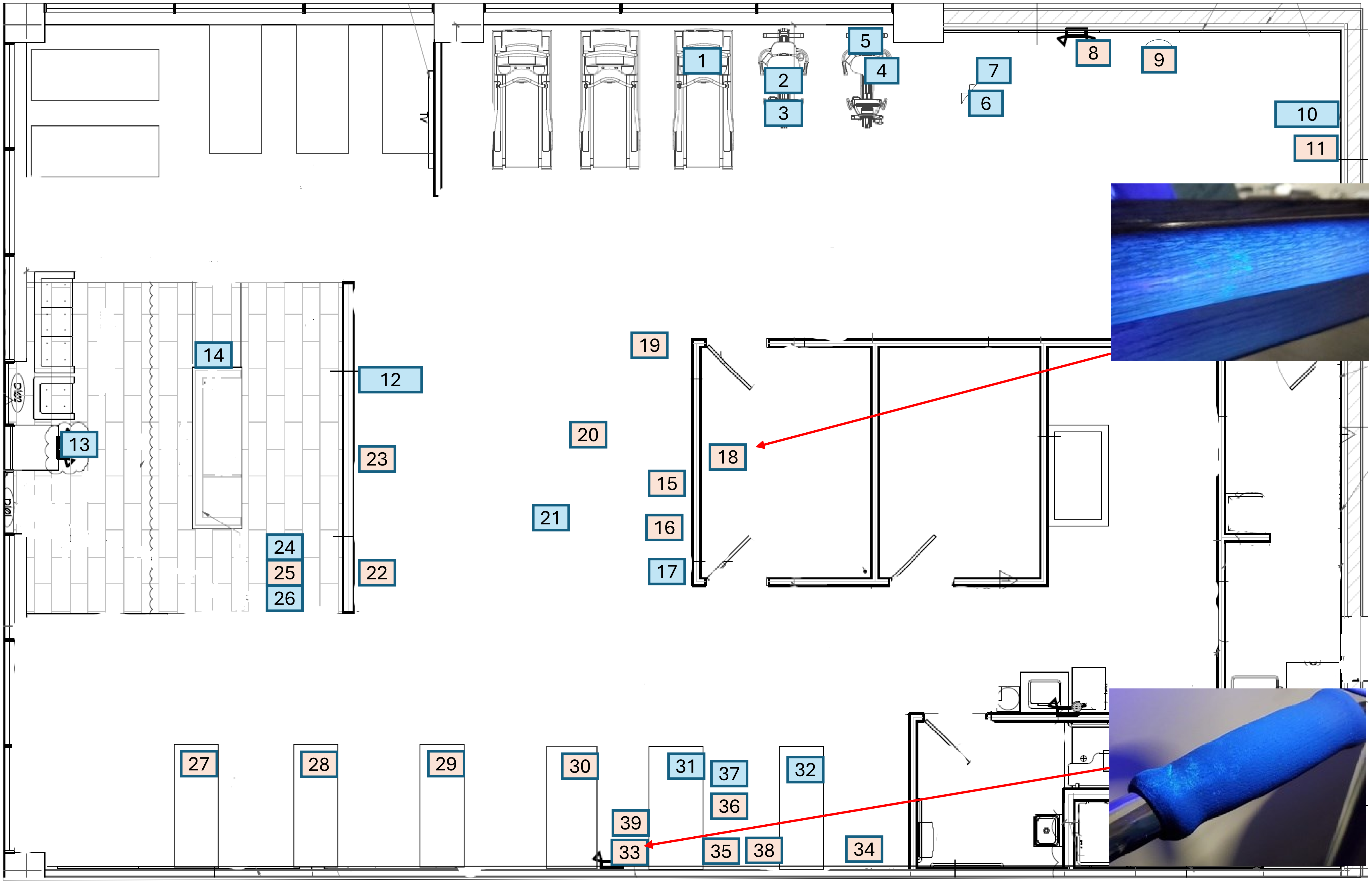

Clinic map showing location and identity of sampled surfaces. Clinic sample numbers correspond with Table 1 in the manuscript. The inset shows two examples of florescent probe identified on surfaces by black light during the sampling process (top, ballet bar; bottom, handle of the step stool).

Data Supplement, Figure S2: Heat Map Comparing Microbiome Distribution Pre- and Post-Cleaning

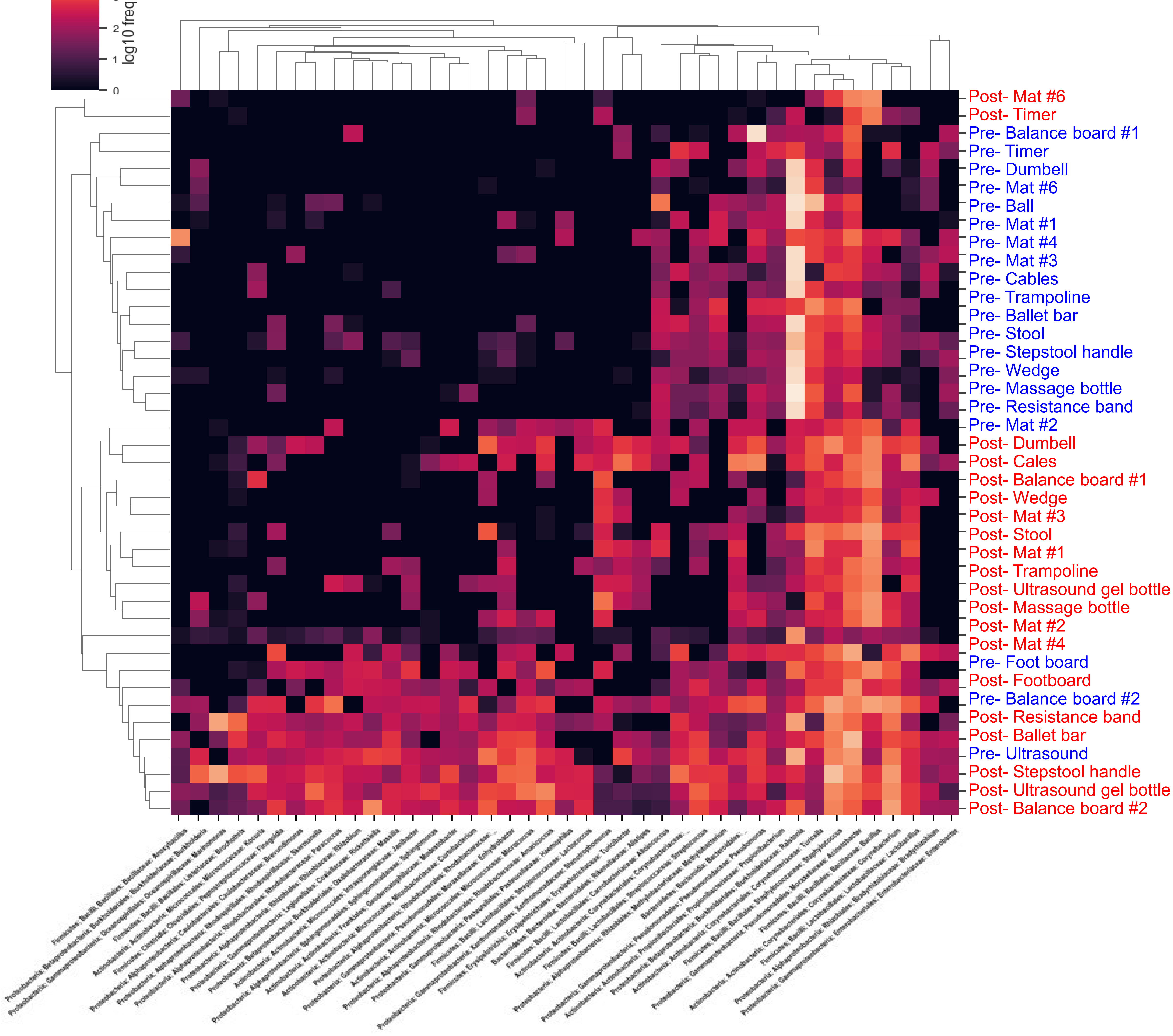

Differential abundance shown as a heatmap of microbiota. Abbreviations are as follows: Post, post-cleaning; Pre, pre-cleaning.

Data Supplement Figure S3: Sample Taxonomy Classifier Heat Map

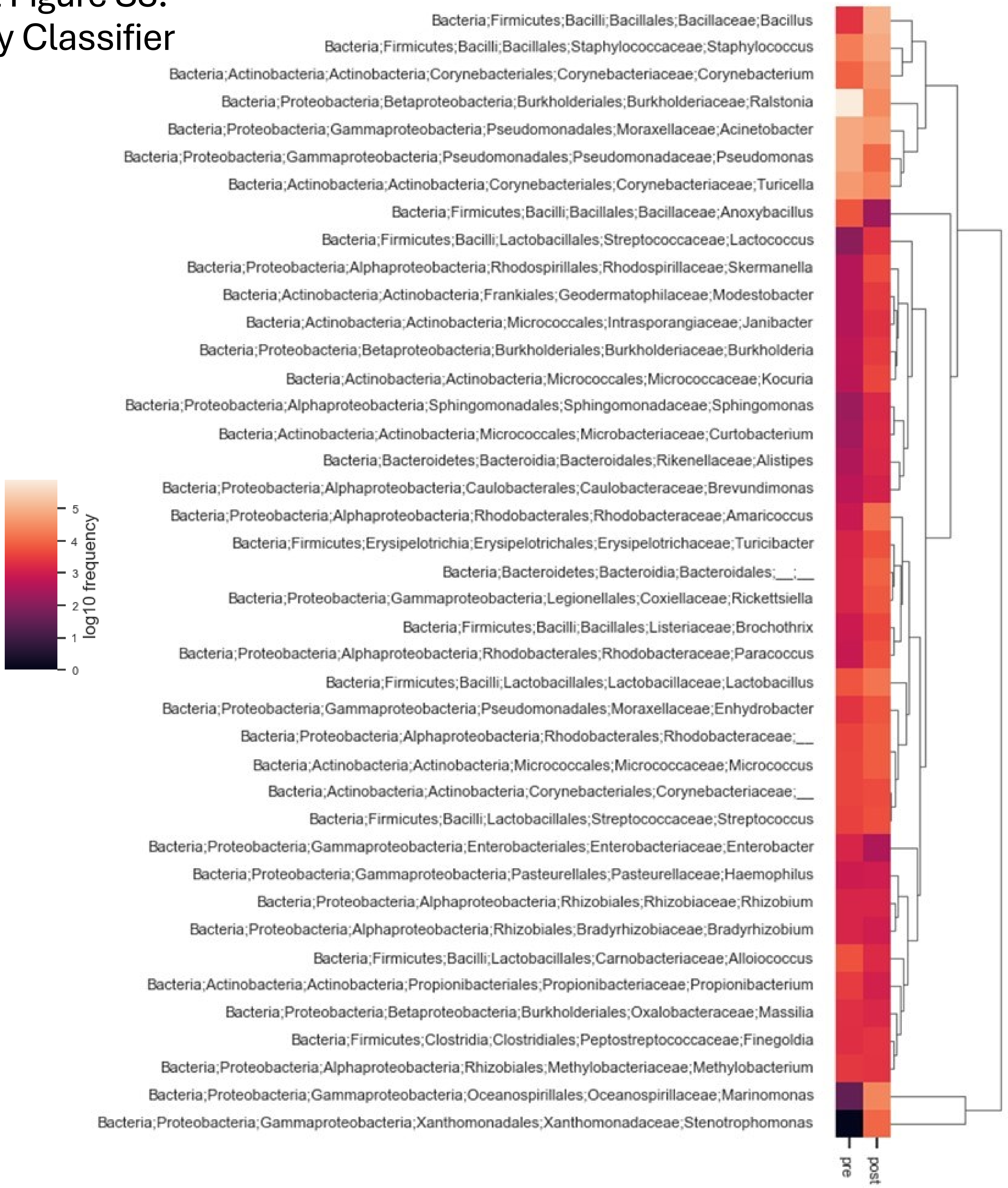

Heatmap showing the phylogenetic relationships of clinic surface bacteria. Abbreviations are as follows: Post, post-cleaning; Pre, pre-cleaning
